# Supplementing Single‐Arm Trials with External Control Arms—Evaluation of German Real‐World Data

**DOI:** 10.1002/cpt.3684

**Published:** 2025-04-16

**Authors:** Martin Russek, Jonas Peltner, Britta Haenisch

**Affiliations:** ^1^ Research Division, Pharmacoepidemiology Federal Institute for Drugs and Medical Devices (BfArM) Bonn Germany; ^2^ Pharmacoepidemiology in Neurodegenerative Disorders German Center for Neurodegenerative Diseases (DZNE) Bonn Germany; ^3^ University of Bonn Center for Translational Medicine Bonn Germany

## Abstract

As single‐arm trials (SATs) are increasingly used in pharmaceutical research, the validity of such study designs needs to be critically assessed. We characterize the feasibility of supplementing SATs with real‐world data (RWD)‐derived external control arms by determining the proportion of SATs on breast cancer and amyotrophic lateral sclerosis (ALS) for which an external control arm based on RWD can be constructed. The main outcome measure is the number and percentage of trials for which all important eligibility criteria and at least one primary endpoint could be identified in one of five German RWD sources. We surveyed all SATs concerning breast cancer or ALS treatment registered in the European Union’s clinical trial registers between 2004 and 2023. Ten out of 379 breast cancer SATs and 2 of 11 ALS SATs could feasibly be supplemented with RWD‐derived external control arms, if all important eligibility criteria and a primary endpoint have to be identifiable in the RWD source. Ninety‐three breast cancer trials had at least one outcome ascertainable in a RWD source, and 35 trials had all important eligibility criteria recorded in a RWD source. Nine ALS trials had at least one primary endpoint ascertainable in RWD sources, and 2 had all important eligibility criteria recorded in a RWD source. Our study shows that SATs with RWD‐derived external control arms will rarely be suitable to establish treatment effects of medicines in the current setting for the investigated phenotypes and that SATs should be designed with limitations of the source of external controls in mind.

**Study Highlights**

**WHAT IS THE CURRENT KNOWLEDGE ON THE TOPIC?**

Regulatory guidance suggests that single‐arm trials should only be considered if alternatives are not feasible. External control arms might improve the interpretability of single‐arm trial results.

**WHAT QUESTION DID THIS STUDY ADDRESS?**

This study evaluates how well external control arms for single‐arm trials in breast cancer and amyotrophic lateral sclerosis can be constructed using 5 different German real‐world data sources.

**WHAT DOES THIS STUDY ADD TO OUR KNOWLEDGE?**

Important eligibility criteria or the primary endpoint were missing for most single‐arm trials. This suggests it is not feasible to construct external control arms for most single‐arm trials when requiring all important eligibility criteria and the primary endpoint to be available in the external data source.

**HOW MIGHT THIS CHANGE CLINICAL PHARMACOLOGY OR TRANSLATIONAL SCIENCE?**

Our findings highlight the importance of designing externally controlled single‐arm trials with the limitations of the external data source in mind.

Single‐arm trials (SATs) have gained popularity in pharmaceutical research over the past years,^1^ due to an increase in focus toward medicines targeted at specific disease subtypes, patients with certain genetic markers, or rare diseases. Despite the drawbacks of SATs compared to randomized controlled trials (RCTs), SATs have their place in establishing drug effects because of, for example, the ethical aspect of placebo treatments in fast‐progressing diseases or increased patient motivation if every trial participant is certain to receive the target treatment rather than a placebo.^2^

However, since SATs do not have a control group, it is much more difficult to distinguish between observed changes due to the treatment and those changes occurring independently of the treatment, for example, due to improved standard of care over time. To facilitate this distinction, one option is to use external control arms (ECAs) derived from real‐world data (RWD). Patients can be matched on relevant variables to increase the comparability of the groups and to reduce the bias introduced by patient selection in the SAT population.

When selecting an external control population, ideally all inclusion and exclusion criteria of the single‐arm trial are applied identically on the RWD population. Additionally, the endpoint of a trial must be available in the RWD source. However, the information about patients in RWD sources is limited and cannot easily be extended. Characterizing primary endpoints and eligibility criteria in SATs and the feasibility of replicating them in RWD sources will help guide researchers to design better studies and inform regulators about the usefulness of requesting SATs to be supplied with external control data.

In this study, we identify eligibility criteria, as well as primary endpoints, used in SATs that would be required for supplementing a RWD‐based ECA. As a case study, we apply this to breast cancer (BC) and ALS SATs in Europe over a period of 20 years. These two disease phenotypes have been selected as examples for RWE use in the pre‐authorization phase in the project Real4Reg,^3^ to cover both a rare disease and one of the most common types of cancer.

## MATERIALS AND METHODS

### Eligibility criteria, information sources and search strategy

We included relevant Phase II‐IV clinical trials registered in one of the two trial registers of the EU, EudraCT^4^ and CTIS^5^ in which all Phase II‐IV trials in the EU need to be registered; EudraCT is the predecessor of CTIS, with optional registration of trials in CTIS starting in 2022 and mandatory registration in 2023. Information on all trials is made publicly available on the public‐facing sections of the registers, from which we extracted the information for this study.

A trial was included in the evaluation if:

(a) It is registered in either EudraCT or CTIS by 31^st^ December 2023,
(b) It is designed or executed as a SAT and
(c) Its patient population involves only BC or ALS patients

We excluded studies that aim to evaluate imaging methods or develop prognostic or predictive models of an outcome. The search string used in the two databases can be found in the **Methods** **S1** in the supplemental material.

In multinational trials, we extracted the information from the protocol for the 1^st^ country in alphabetical order for which the trial information is available in either English or German. If no trial information is available in those languages, we used the translation application DeepL^6^ to translate into English.

We evaluated the feasibility of ECA supplementation based on 5 different German RWD sources, selected on the basis of sample size and public availability of metadata. The RWD sources selected were billing data of the statutory health insurance providers in Germany,^7^ an epidemiological cancer registry,^8^ a clinical cancer registry,^9^ an ALS registry,^10^ and electronic health record data.^11^ A description of the data sources is given in **Table** **S1** in the **Methods** **S1**.

### Study records

All relevant information for included trials was downloaded from the public‐facing areas of the two registers using the R package ctrdata.^12^ In a first step, we screened all trials included in CTIS for duplicate entries, and if relevant, deleted those entries from the list of EudraCT trials. The remaining trials were screened for inclusion independently by two researchers.

The data items retrieved from the registers are listed in the **Methods** **S1**. Trial eligibility criteria, as well as outcome measures, were categorized into pre‐specified super‐categories to allow for efficient assessment of replication feasibility. Super‐categories were created by reviewing eligibility criteria in an arbitrarily selected set of 8 randomized breast cancer trials and 6 randomized ALS trials, with post‐hoc additions if a criterion did not fit any of the pre‐specified categories. Categorization was performed independently by two researchers.

### Main outcome measure

To evaluate the feasibility of replicating the trial eligibility criteria of the given SATs, we graded each identified eligibility criterion category via the two domains of availability and importance. Information on the grading with examples is given in **Table** **S2** in the **Methods** **S1**.

Availability refers to the presence of data on each eligibility category in the relevant data sources. The levels of availability are:

(1) Data directly available
(2) Can be approximated with available data
(3) Not feasible to approximate

Importance refers to the potential impact the presence/absence of data on a specific eligibility category has on analysis results. The levels of importance are:

(1) Without impact on research question
(2) Conservative estimation possible
(3) Crucial for research question

As availability of the primary endpoint in RWD sources is crucial for the supplementation of an external control arm, the evaluation is limited to one dimension with the following levels:

(1) Exactly available
(2) Conservative estimation possible
(3) Not available

Supply of an external RWD‐based control arm was judged to be feasible if there are no eligibility criteria having both importance and availability level 3 and at least one primary endpoint having availability levels 1 or 2. The full evaluation of availability and importance is given in **Tables** **S3** and **S4**, and definitions for each eligibility criterion category are given in the **Methods** **S1**.

### Other variables of interest

For all trials, we characterized the trial phase (Phase II, III or IV), trial status (e.g., ongoing, completed), status of sponsor (e.g., commercial, academic), date of record entry, whether the SAT is an extension to a previous trial, and, if applicable, competent authority decision, end of trial status, and the corresponding decision and end of trial dates.

### Statistical analysis

We used descriptive statistics, characterizing frequencies of eligibility criteria used, frequency of outcome measures used, and shares of trials for which RWD‐based external control arms can be feasibly supplied. As supplementary analysis following the methodology of Bartlett et al.,^13^ we describe which share of eligibility criteria can be replicated in RWD sources.

Tables and plots were generated in R,^14^ using the packages openxlsx^15^ and ggplot2.^16^

## RESULTS

**Figure 1:**
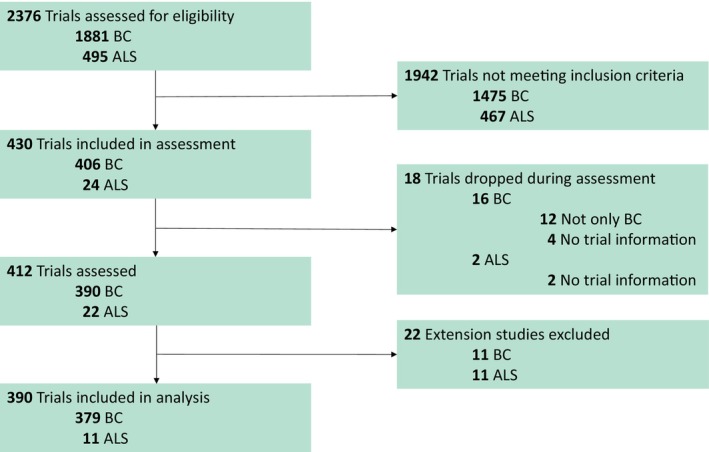
Study flowchart.

In the years 2004–2023, 412 SATs concerning breast cancer or ALS patients were registered in the EU, including 22 (11 BC, 11 ALS) extension studies, that is, studies providing patients with treatment medication after completion of a previous trial. We excluded those trials from our study, as the trial eligibility criteria were not well described and referenced the original trials without enough identification details. See **Figure** **1** for the study flowchart.

**Figure 2:**
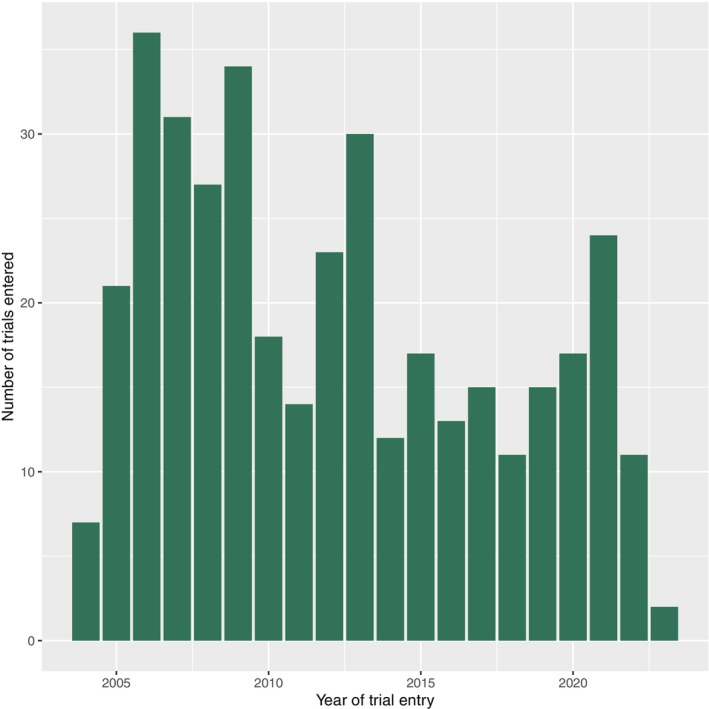
BC studies by year of registration.

Of the remaining 390 trials included in the final evaluation, 379 trials were BC SATs and 11 ALS SATs. Out of the BC SATs, 219 (57.8%) were registered by non‐commercial sponsors, 190 (50.1%) were completed, 61 (16.0%) ended prematurely, 1 (0.3%) was temporarily halted, and 122 (32.1%) were still ongoing. Four trials (1.1%) executed solely in Great Britain were not followed in EU registers after Great Britain left the EU. Of the BC SATs, 342 (90.2%) were Phase II (Therapeutic exploratory) trials, 16 (4.2%) Phase III (Therapeutic confirmatory) trials, and 18 (4.7%) Phase IV (Therapeutic use) trials. **Figure** **2** shows the distribution of SATs by year, indicating a slight downward trend in the number of BC SATs registered in the EU.

**Figure 3:**
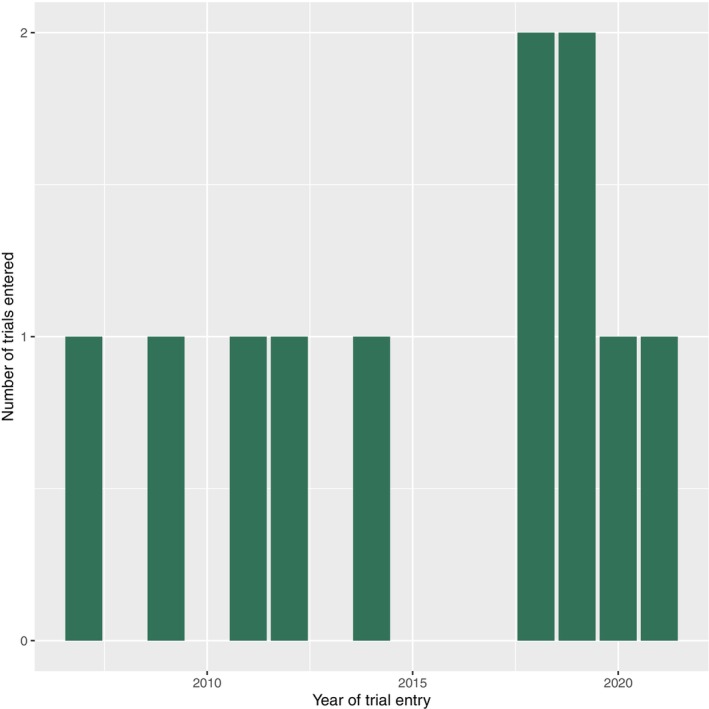
ALS studies by year of registration.

Out of the 11 ALS SATs, 4 (36%) were registered by non‐commercial sponsors, 7 (64%) were completed, 2 (18%) ended prematurely, 1 (9%) was ongoing, and 1 (9%) was not followed up after Great Britain leaving the EU. There were 9 (82%) Phase II trials, 1 (9%) Phase III trial, and 1 Phase IV trial. **Figure** **3** shows the distribution of ALS SATs by year, indicating a slight increase in the number of trials in recent years.

### Inclusion and exclusion criteria

Of the 379 BC SATs, 248 (65.4%) trials had restrictions on participants’ gender – 247 trials (65.2%) included only female patients, 1 trial (0.3%) only male patients. One trial included patients below 18 years of age. The 3 most common BC‐specific criteria were restrictions on the tumors’ HER2, estrogen receptor (ER) or progesterone receptor (PR) status. Two hundred eighty‐three trials (74.7%) had restricted participants’ HER2 status, 183 (48.3%) the ER status, and 151 (39.8%) the PR status. In total, 319 trials (84.2%) required a certain performance status such as the Eastern Cooperative Oncology Group (ECOG) performance status, and 364 trials (96.0%) had restrictions on prior or concurrent medications (excluding the trial medication).

None of the 11 ALS SATs had restrictions on a participant’s gender or included patients below 18 years of age. One trial (9%) had restrictions on participants’ ALS family history, 4 trials (36%) had requirements on ALS‐related performance scores, and 7 trials (63%) required other psychological testing outside routine care. Seven trials (63%) also had a limitation on a participant’s time since the onset of ALS symptoms.

The full table of eligibility criteria categories, as well as primary endpoints per trial, can be found in **Table** **S5**.

### Endpoints

A full table of primary endpoint counts is given in **Tables** **S6** and **S7**. Two hundred fifty‐two of the BC SATs (66.5%) used Response Evaluation Criteria in Solid Tumors (RECIST) based rates and 47 (12.4%) Time‐to‐event endpoints based on RECIST. Eighteen trials (4.7%) used occurrence of specific adverse events (AEs) as endpoints, 12 of which would be routinely diagnosed. Thirty‐five trials (9.2%) registered occurrence of AEs or safety as primary endpoints without further specification. Survival was a primary endpoint for 7 trials (1.8%). Fifty‐four BC SATs (14.2%) were registered with more than one primary endpoint.

Five of the 11 ALS SATs (46%) registered AEs or safety without further specification as a primary endpoint, 4 (36%) listed specific AEs, two of which (18%) would be routinely diagnosed. Seven trials (46%) had laboratory or vital measurements as a primary endpoint. Survival was an endpoint for 2 trials (18%). Nine out of the 11 ALS SATs (82%) were registered with more than one primary endpoint.

### Availability of inclusion and exclusion criteria and primary endpoints

Out of the 379 BC SATs, 10 trials (2.6%) would be suitable for EHR‐based ECAs, if all important eligibility criteria and a primary endpoint have to be available, 2 (0.5%) for ECAs based on claims data and none would be suitable for ECAs based on epidemiological or clinical cancer registries. The eligibility criteria of 35 trials (9.2%) could be replicated in EHR data, 4 (1.1%) in claims data and 1 (0.3%) in clinical and epidemiological cancer registers. Ninety trials (23.7%) had at least one primary endpoint that could be identified in EHR data, 51 (13.5%) in claims data and 50 (13.2%) in clinical or epidemiological cancer registries. A hypothetical linkage of the 4 RWD sources would allow for suitable ECAs for 17 trials (4.4%) and could replicate the eligibility criteria of 70 trials (18.4%) and allow ascertainment of at least one primary endpoint of 93 trials (24.5%).

Of the 11 ALS SATs, one (9%) would be suitable for an EHR‐based ECA, two (18%) would be suitable for claims‐based ECAs, and none for ECAs based on an ALS registry. For two (18%) trials, all eligibility criteria can be validly replicated in EHRs and claims data, and for none in the ALS registry. Eight trials (73%) have at least one primary endpoint that could be ascertained in EHR data, 9 (82%) in claims data, and 3 (27%) in the ALS registry. A hypothetical linkage between all 3 data sources would not improve feasibility compared to claims data alone.

In a post‐hoc exploratory analysis for EHR‐based ECAs in BC SATs, we see that the feasibility is slightly higher for Phase III and IV trials (8.8%, *n* = 3) compared to Phase II trials (2%, *n* = 7), and there are no substantial differences between years of entry and between sponsor types (commercial vs. non‐commercial). See **Table** **S8** for a detailed description.

**Figure 4:**
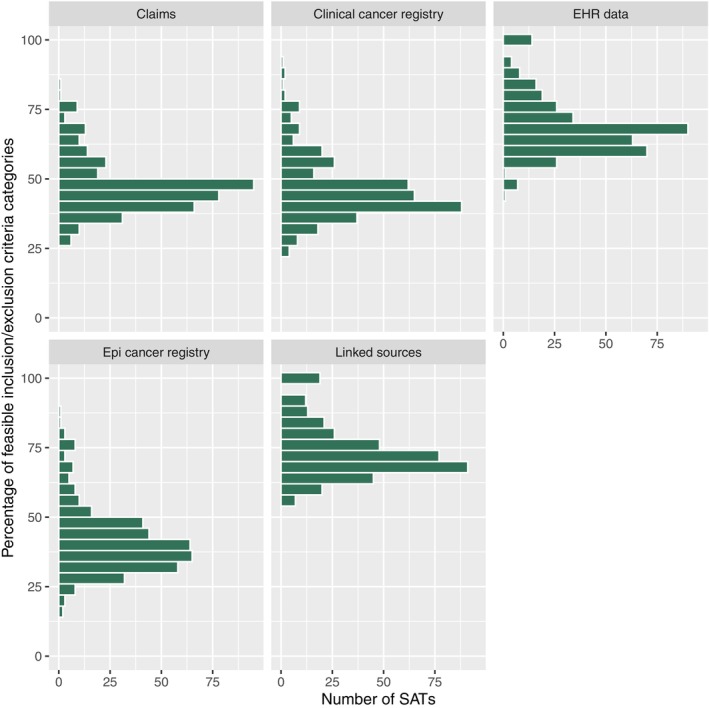
Histograms of BC SATs, by the percentage of eligibility criteria categories that could be identified in the respective RWD source.

**Figure 5:**
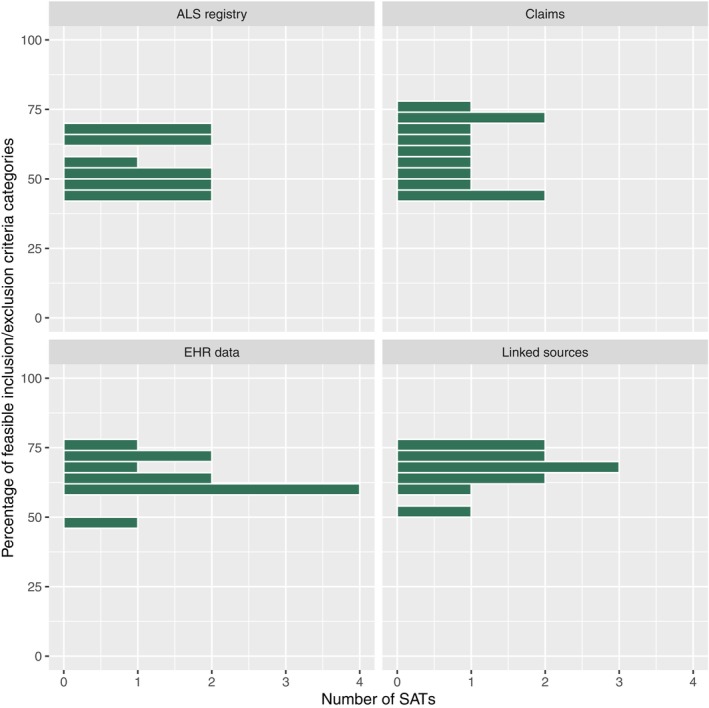
Histograms of ALS SATs, by the percentage of eligibility criteria categories that could be identified in the respective RWD source.

**Figures** **4** and **5** show the results following the methodology of Bartlett et al.^13^ The figures display what percentage of each SAT’s eligibility criteria could be identified in RWD sources, regardless of the importance of the criteria. We see that for EHR data, a considerable number of BC SATs have more than 80% of their eligibility criteria categories available, while most trials stay below 80% for the other data sources in both ALS and BC trials.

## DISCUSSION

We found that few breast cancer and ALS single‐arm trials we identified were suitable for RWD‐based external control arms, if requiring that all important eligibility criteria and a primary endpoint of the SATs are available in the RWD source. This highlights the massive challenges of supplementing SATs with RWD‐based ECAs, as has been described previously, for example, by the FDA as follows: *in many situations*, *however*, *the likelihood of credibly demonstrating the effectiveness of a drug of interest with an external control is low*.^17^

In a similar study to ours,^13^ Bartlett et al. found that only around 15% of RCTs investigated allowed for replication of their eligibility criteria and endpoints in RWD sources. Another study found that none of the 50 post‐approval confirmatory clinical trials in the United States could be emulated using RWD.^18^ Our results finding a lower share than that in Bartlett et al. has at least two major reasons.

The first reason is the nature of SATs compared to RCTs. The randomization aspect of RCTs enables identification of the treatment effect by diminishing all outside influences on outcome development when comparing between the treatment and control groups. While traditionally, RCTs are still relatively restrictive with respect to their participant populations, they can allow for rather large heterogeneity among participants. Isolating the treatment effect in a SAT is very challenging, as was highlighted in a draft reflection paper by the European Medicines Agency.^19^ One of the techniques to isolate the treatment effect is to homogenize the trial population as much as feasible. This naturally requires more stringent eligibility criteria, which diminishes the likelihood of all criteria being recorded in RWD.

Secondly, Bartlett et al. judged a trial to be replicable if 80% of its eligibility criteria and at least one endpoint were identified in RWD. While we use the same condition for the endpoint, our criterion for eligibility criteria is more restrictive, as we do not allow any important criterion missing in RWD sources. We see in **Figures** **4** and **5** that feasibility would improve for BC using the metric of Bartlett et al.

Previous studies have also highlighted other challenges of SATs with ECAs. When attempting to use an EHR‐derived ECA, a large number of missing baseline information may be found,^20^ which is a challenge we have not incorporated into our analysis. Since SATs are frequently proposed because of small sample sizes, having a large share of information missing might not only bias results but also reduce the utility of smaller RWD sources. Another review of trials with ECAs found a major lack of methodological rigor when utilizing external controls,^21^ with most trials not employing any form of matching or weighting of patients between the trial population and external control.

Studies attempting to calibrate ECAs to RCT arms have found mixed results. While some studies have found similar overall survival between the comparator arm of an RCT and that of an RWD‐based ECA,^22^, ^23^ other studies have observed overall survival estimates being higher in trial populations than in matched ECA populations.^24^, ^25^ Interestingly, most of the calibration studies concerned non‐small cell lung cancer, indicating that even within the same phenotype, the validity of ECAs cannot easily be judged.

Despite all challenges of complementing SATs with RWD‐derived ECAs, there are still use cases where this study design can be useful. A review of drug marketing authorization applications (MAAs) found that 79% (27 of 34) of MAAs based on non‐randomized studies (most of which were single‐arm or multi‐arm uncontrolled trials) with external controls as primary evidence gained EU approval,^26^ highlighting that high‐quality studies are valuable in establishing therapeutic effects of medicines.

There are several ways to improve the evidence value of SATs with ECAs. Our study highlighted a major one: Designing the original trial with the ECA in mind. The definition of eligibility criteria as well as endpoints should be chosen such that both clinical relevance and patient benefit are maintained and that a valid comparison with RWD‐based ECAs is possible. To ensure this, regulators regularly highlight the importance of early interactions during the trial design stage.^27^ The validity of comparisons between trial participants and ECA subjects depends on a variety of factors, including data quality, rigorous study design, appropriate analytic methods, and adherence to good procedural practices. Potential improvements to study design validity include utilization of matching or weighting methods such as inverse probability of treatment weighting^28^ to establish comparable study groups,^29^ hybrid ECAs incorporating trial participants,^25^ and quantitative bias analysis for external control arms.^30^ On a global level, improving the quality and quantity of information captured in RWD sources, for example, by linkage of different data sources and variables, would also improve research and decision‐making involving RWD considerably.

For the contextualization of our study, it is important to consider that for our assessment of feasibility, we only considered replicability of eligibility criteria and primary endpoints of SATs. There may often be other variables that would be crucial to collect for successful participant matching, such as smoking status. We have also not considered methodological challenges related to effect estimation, such as the challenge of defining time zero in time‐to‐event outcomes^19^ or the sample size remaining after applying all eligibility criteria.

While we investigated two phenotypes with distinct characteristics—a rare disease with limited treatment options and one of the most common forms of cancer, with a broad range of available treatments—there might be other fields in which RWD‐based ECAs are more feasible for SATs. We saw that survival and occurrence of AEs were commonly available in RWD sources. SATs with those endpoints, as well as some other endpoints like time‐to‐next‐treatment^31^ could be more suitable for being supplemented with RWD‐based ECAs. The same is true for phenotypes where trials have less restrictive eligibility criteria. That could include medications being targeted to the whole disease population rather than populations defined using molecular analysis or by disease stage, as we have often seen with BC.

Future research on the topic of external control arms could extend our evaluation to more disease phenotypes and other real‐world data sources. By considering both a rare disease and one of the most common types of cancer globally, we hope to get a relatively good contrast, but it is possible that SATs for other phenotypes typically have less strict eligibility criteria and ECA supplementation would be more feasible.

While conducting SATs with RWD‐based ECAs is an appealing option in certain situations, our results indicate that this option might not be feasible for many SATs, including most BC and ALS trials, taking into account all eligibility criteria of SATs. Limitations in the depth of data mean that endpoints and important eligibility criteria often cannot be determined in the German RWD sources examined. This shows that those limitations should already be considered when designing the SAT.

## FUNDING

The research leading to these results has received funding from the European Community’s Horizon Europe Programme under grant agreement No. 101095353 (Real4Reg). The funder did not play a role in study design, execution, or interpretation.

## CONFLICT OF INTEREST

The authors declared no competing interests for this work.

## AUTHOR CONTRIBUTIONS

All authors wrote the manuscript; M.R. and B.H. designed the research; M.R. and J.P. performed the research; M.R. analyzed the data.

## Supporting information


Data S1.

Data S2.

## References

[1] Sasinowski, F.J. , Panico, E.B. & Valentine, J.E. Quantum of effectiveness evidence in FDA’s approval of orphan drugs: update, July 2010 to June 2014. Ther Innov Regul Sci 49, 680–697 (2015). 30227043 10.1177/2168479015580383

[2] Subramaniam, D. , Anderson‐Smits, C. , Rubinstein, R. , Thai, S.T. , Purcell, R. & Girman, C. A framework for the use and likelihood of regulatory acceptance of single‐arm trials. Ther Innov Regul Sci 58, 1214–1232 (2024). 39285061 10.1007/s43441-024-00693-8 PMC11530569

[3] Peltner, J. *et al*. The EU project Real4Reg: unlocking real‐world data with AI. Health Res Policy Syst 23, 27 (2025). 40016823 10.1186/s12961-025-01287-y PMC11869640

[4] EudraCT Public website – Home page <https://eudract.ema.europa.eu/>. Accessed December 14, 2023.

[5] EMA . Clinical Trials in the European Union <https://euclinicaltrials.eu/>. Accessed December 14, 2023.

[6] DeepL Übersetzer: Der präziseste Übersetzer der Welt <https://www.deepl.com/translator>. Accessed December 14, 2023.

[7] Forschungsdatenzentrum Gesundheit . Datensatzbeschreibung des Forschungsdatenzentrums Gesundheit (2024) 10.5281/zenodo.11472236.

[8] Meisegeier, S. , Imhoff, M. , Berg, K. & Kraywinkel, K. Bundesweiter klinischer Krebsregisterdatensatz – Datenschema und Klassifikationen (2023) 10.5281/zenodo.10022040.

[9] Über uns . Klinisches Krebsregister Niedersachsen <https://kk‐n.de/klinisches‐krebsregister/>. Accessed July 18, 2024.

[10] Nagel, G. , Ünal, H. , Rosenbohm, A. , Ludolph, A.C. & Rothenbacher, D. Implementation of a population‐based epidemiological rare disease registry: study protocol of the amyotrophic lateral sclerosis (ALS) – registry Swabia. BMC Neurol 13, 22 (2013). 23414001 10.1186/1471-2377-13-22 PMC3582473

[11] Lingner, H. *et al*. Health science research with primary care routine data from electronic patient records: the BeoNet registry. Gesundheitswesen 80, 1026–1034 (2018). 28697524 10.1055/s-0043-108544

[12] Herold, R. ctrdata: Retrieve and Analyze Clinical Trials in Public Registers <https://CRAN.R‐project.org/package=ctrdata> (2023).

[13] Bartlett, V.L. , Dhruva, S.S. , Shah, N.D. , Ryan, P. & Ross, J.S. Feasibility of using real‐world data to replicate clinical trial evidence. JAMA Netw Open 2, e1912869 (2019). 31596493 10.1001/jamanetworkopen.2019.12869 PMC6802419

[14] R Core Team . R: A Language and Environment for Statistical Computing (R Foundation for Statistical Computing, Vienna, Austria, 2023) https://www.R‐project.org/.

[15] Schauberger, P. & Walker, A. Openxlsx: Read, Write and Edit Xlsx Files <https://CRAN.R‐project.org/package=openxlsx> (2023).

[16] Wickham, H. ggplot2: Elegant Graphics for Data Analysis (Springer‐Verlag, New York, 2016) <https://ggplot2.tidyverse.org>.

[17] Center for Drug Evaluation and Research, US Food and Drug Administration . Considerations for the Design and Conduct of Externally Controlled Trials for Drug and Biological Products <https://www.fda.gov/regulatory‐information/search‐fda‐guidance‐documents/considerations‐design‐and‐conduct‐externally‐controlled‐trials‐drug‐and‐biological‐products> (2023). Accessed June 20, 2024.

[18] Wallach, J.D. *et al*. Feasibility of using real‐world data to emulate Postapproval confirmatory clinical trials of therapeutic agents granted US Food and Drug Administration accelerated approval. JAMA Netw Open 4, e2133667 (2021). 34751763 10.1001/jamanetworkopen.2021.33667 PMC8579227

[19] Single‐arm trials as pivotal evidence for the authorisation of medicines in the EU | European Medicines Agency <https://www.ema.europa.eu/en/news/single‐arm‐trials‐pivotal‐evidence‐authorisation‐medicines‐eu>. Accessed June 20, 2024.

[20] Rudrapatna, V.A. *et al*. Creation of an ustekinumab external control arm for Crohn’s disease using electronic health records data: a pilot study. PLoS One 18, e0282267 (2023). 36862717 10.1371/journal.pone.0282267 PMC9980824

[21] Zayadi, A. *et al*. Use of external control arms in immune‐mediated inflammatory diseases: a systematic review. BMJ Open 13, e076677 (2023). 10.1136/bmjopen-2023-076677 PMC10729249 38070932

[22] Walker, B. , Ray, H.E. , Shao, P. , D’Ambrosio, C. , White, C. & Walker, M.S. Comparing prospectively assigned trial and real‐world lung cancer patients. J Comp Eff Res 13, e230176 (2024). 38785683 10.57264/cer-2023-0176 PMC11225159

[23] Yin, X. , Mishra‐Kalyan, P.S. , Sridhara, R. , Stewart, M.D. , Stuart, E.A. & Davi, R.C. Exploring the potential of external control arms created from patient level data: a case study in non‐small cell lung cancer. J Biopharm Stat 32, 204–218 (2022). 34986069 10.1080/10543406.2021.2011901

[24] Børø, S. , Thoresen, S. , Boge Brant, S. & Helland, Å. Initial investigation of using Norwegian health data for the purpose of external comparator arms – an example for non‐small cell lung cancer. Acta Oncol 62, 1642–1648 (2023). 37801361 10.1080/0284186X.2023.2264484

[25] Struebing, A. *et al*. Augmenting external control arms using Bayesian borrowing: a case study in first‐line non‐small cell lung cancer. J Comp Eff Res 13, e230175 (2024). 38573331 10.57264/cer-2023-0175 PMC11036906

[26] Goring, S. *et al*. Characteristics of non‐randomised studies using comparisons with external controls submitted for regulatory approval in the USA and Europe: a systematic review. BMJ Open 9, e024895 (2019). 10.1136/bmjopen-2018-024895 PMC6398650 30819708

[27] Khachatryan, A. , Read, S.H. & Madison, T. External control arms for rare diseases: building a body of supporting evidence. J Pharmacokinet Pharmacodyn 50, 501–506 (2023). 37095406 10.1007/s10928-023-09858-8 PMC10673956

[28] Robins, J.M. , Hernán, M.Á. & Brumback, B. Marginal structural models and causal inference in epidemiology. Epidemiology 11, 550–560 (2000). 10955408 10.1097/00001648-200009000-00011

[29] Hashmi, M. , Rassen, J. & Schneeweiss, S. Single‐arm oncology trials and the nature of external controls arms. J Comp Eff Res 10, 1052–1066 (2021). 34156310 10.2217/cer-2021-0003

[30] Gray, C. *et al*. Use of quantitative bias analysis to evaluate single‐arm trials with real‐world data external controls. Pharmacoepidemiol Drug Saf 33, e5796 (2024). 38680093 10.1002/pds.5796

[31] Campbell, B.A. , Scarisbrick, J.J. , Kim, Y.H. , Wilcox, R.A. , McCormack, C. & Prince, H.M. Time to next treatment as a meaningful endpoint for trials of primary cutaneous lymphoma. Cancer 12, 2311 (2020). 10.3390/cancers12082311 PMC7463470 32824427

