# Supplementary material for: Supplementing Single‐Arm Trials with External Control Arms—Evaluation of German Real‐World Data
Source: Clin Pharmacol Ther. 2025 Apr 16;118(6):1443–50. doi: 10.1002/cpt.3684 (PMC12641071; doi:10.1002/cpt.3684)
Supplement: Supplementary file 1 — Data S1. [file CPT-118-1443-s001.docx]

Content

[Search string 2](#_Toc192060491)

[Data items downloaded from trial registries 2](#_Toc192060492)

[Trial inclusion/exclusion criteria categorization 2](#_Toc192060493)

[Trial inclusion/exclusion criteria categorization 5](#_Toc192060494)

[Tables 6](#_Toc192060495)

## Search string

Tight search:

(“single-arm” OR (“single arm”) OR non-randomized OR platform OR pilot) AND

(((breast OR mammary) AND (cancer OR neoplasm OR carcinoma OR tumor OR tumour))

(“single-arm” OR (“single arm”) OR non-randomized OR platform OR pilot) AND

(ALS OR “amyotrophic lateral sclerosis” OR Charcot OR Gehrig OR Gehrig’s OR Lou-Gehrigs))

Broad search:

(((breast OR mammary) AND (cancer OR neoplasm OR carcinoma OR tumor OR tumour))

(ALS OR “amyotrophic lateral sclerosis” OR (Charcot OR Gehrig OR Gehrig’s OR Lou-Gehrigs)))

## Data items downloaded from trial registries

- Title
- Link to registration
- Trial phase
- Trial Status (e.g. completed)
- Medical condition (MedDRA)
- Population age (e.g. adult, elderly)
- Gender
- Status of sponsor (e.g. commercial)
- Marketing authorization status pre-trial
- Principal inclusion criteria, categorized from free text
- Principal exclusion criteria, categorized from free text
- Primary endpoint
- Date of record entry
- If applicable: Competent Authority Decision
- If applicable: Date of Competent Authority Decision
- If applicable: End of Trial Status
- If applicable: Date of the global end of the trial

## Trial inclusion/exclusion criteria categorization

Age: Requirements on a participant’s age

Gender_male: Trials restricted to male subjects

Gender_female: Trials restricted to female subjects

Race: Requirements on a participant’s race

Contraception: Any requirements on the participants to make use of contraception of any type or effectiveness including abstinence

Childbearing potential: Requirements on a participant’s potential to become pregnant. If restriction applied to use of contraception, only contraception is recorded. Including participants wishing to preserve ovarian function

Pregnancy: Restrictions on participants’ pregnancy status

Breastfeeding: Restrictions on participants breastfeeding or lactating

Menopause: Requirements on participants’ menopausal status. If restriction applied to use of contraception, only contraception is recorded

Informed consent: Requirement of a signed informed consent form

Laboratory sample availability: Requirements on availability of laboratory samples including tumour biopsies, blood samples, imaging etc. before trial entry

Ability to take in treatment: Requirements on participants’ ability to take in trial medication (e.g. to swallow pills), ability to follow trial instructions, ability to comprehend informed consent form, or hypersensitivity to treatment compounds

Insurance status: Requirements on participant to be insured

Relation to sponsor: Explicit exclusion of employees of trial sponsor, relatives of study personnel, individuals involved in the trial design etc.

BC general: Only recorded if no other breast cancer related criteria present

BC with other treatment infeasible: Resistance to other medication, unresectable tumours etc.

BC metastatic: Requirements on breast cancer metastases, including presence of specific metastases

Laterality_location: Requirements on a tumour’s location within the breast, restrictions to uni-/bilateral BC or the number of tumour foci

HER2: Restrictions on a patient’s HER2 (or ErbB2) receptor status. All receptor statuses include requirement of known receptor status without restriction on the status.

ER: Estrogen receptor status

PR: Progesterone receptor status

FGFR1: Fibroblast growth factor receptor 1 status

AR: Androgen (or NR3C4) receptor status

PRLR: Prolactin receptor status

BRCA1 or BRCA2 mutation: Restrictions on BRCA1 or BRCA2 gene mutations

p53 mutation: Restrictions on p53 gene mutations. p53 expression is categorized to histochemically defined subtype.

ErbB2 mutation: Restrictions on ErbB2 mutations. ErbB2/HER2 expression is categorized to HER2.

BC with other genetic mutations: Any restriction on genetic mutations related to BC, e.g. PALB2 gene mutation.

Histopathology-defined subtype: Restrictions on BC subtypes characterized by histopathology, e.g. apocrine or lobular BC

Symptom-defined subtype: Restrictions on BC subtypes defined by symptoms, e.g. inflammatory BC

miRNA-defined subtype: Restrictions on BC subtypes defined by miRNAs, e.g. let-7, miR-155

Histochemically defined subtype: Restrictions on BC subtypes defined histochemically, e.g. via ki67 presence or p53 expression

ECOG or similar performance scores: Restrictions on participants’ disease severity scores, e.g. ECOG, ALS-related scores or degrees of independence

RECIST_staging_tumor size: Restrictions based on RECIST, tumour stage or tumour size

Residual disease: Restrictions on the presence of disease after previous treatment

Life expectancy: Restrictions on a participant’s expected remaining survival time

Organ function: Restrictions on proper function of certain organs, if no explicit tests are specified

Time since symptom onset: Restrictions on a patient’s time since symptoms have been noticed or recorded, including disease duration

Treatment history general: Restrictions on prior/concurrent treatment with authorized medicines, radiation etc. Only recorded if not belonging to one of the following categories

Unauthorized medicines: Restrictions on use of medicines without regulatory approval and not part of a clinical trial

Vaccination: Restrictions on prior vaccinations

Experimental drugs or other trial participation: Restrictions on prior/concurrent participation in other clinical trials

Surgery: Restrictions on prior surgeries or surgical interventions

Disease history general: Restrictions on participants’ prior or concurrent diseases, including drug/alcohol abuse, not including BC or ALS

Disease history graded: Restrictions on the grade of a participant’s prior/concurrent disease, not included BC/ALS. E.g. NYHA class for heart failure

Disease history uncontrolled: Exclusion of participants with uncontrolled concurrent diseases, excluding BC/ALS

Disease history through laboratory testing: Restrictions on participant’s health status based on tests performed during screening, excluding BC/ALS. E.g. calculated creatinine clearance or Left-ventricular ejection fraction.

Weight_BMI: Restrictions on a patient’s weight or BMI. If weight is part of measurements like calculated creatinine clearance, then recorded as ‘Disease history through laboratory testing’

Other genetic mutations: Restrictions on a patient’s genetic mutations not related to BC

Psychological assessment outside routing care: Restrictions on patients through psychological tests outside of ALS/BC

Family history: Restrictions on patients with/without a family history of BC/ALS

ALS general: Only recorded if no specific ALS types required

Sporadic ALS: Restriction to patients with sporadic ALS

Familial ALS: Restriction to patients with familial ALS

Initial-site-derived subtype: Restrictions on ALS subtypes based on the site of onset, e.g. lower, bulbar, or limb-onset

Neuron-type-derived subtype: Restrictions on ALS subtypes based on type of neurons affected, e.g. Primary lateral sclerosis (PLS)

Omics-derived subtype: Restrictions on ALS subtypes based on omics, e.g. Transcriptomal dysregulation ALS (ALS-TD)

Age-of-onset-derived subtype: Restrictions on ALS subtypes based on the age of onset, e.g. juvenile ALS

## Trial inclusion/exclusion criteria categorization

RECIST ORR_CBR_CR_PR_SD: Rates of events based on RECIST, evaluated at a single time point

RECIST TTE: Time-to-event endpoints based on RECIST, evaluated continuously. Including endpoints combining RECIST and other events, like Progression-free survival.

Survival: Death/survival as an endpoint

Change in tumour size: Change of size of the primary tumour, not based on RECIST

AE_safety general: Safety as primary endpoint, without pre-specified adverse events

Specific AEs routine: Occurrence of specific AEs as primary endpoint, excluding AEs not routinely assessed or AEs of certain grade

Specific AEs Non-routine testing: Occurrence of specific AEs that need targeted testing for detection

Specific AEs graded: Specific AEs of certain severity

Other tolerability: Any tolerability endpoints not covered by previous categories

Laboratory measurements: Laboratory measurements such as ki67 concentration as primary outcome

Vital measurements: Physical measurements (not including laboratory measurements) as primary outcome, e.g. Forced Vital Capacity

ECG_Imaging: Outcomes based on Imaging or ECG data, excluding RECIST-based evaluations

ALS-related scores: Performance scores related to ALS as primary endpoint

Psychological scores: Psychological scores not primarily designed for ALS as primary endpoint

VAFS_Time to intervention: Time-to-event endpoints where the event is an intervention rather than an outcome

PROs: Patient related outcomes as primary endpoint

Treatment discontinuation: Rate of discontinuation or Time to discontinuation as primary endpoint

Tables

| Data source type | Name | Description |
| --- | --- | --- |
| Claims Data | Forschungsdatenzentrum Gesundheit (Health Data Lab, HDL) | The HDL contains reimbursement data from all statutory German health insurance companies, containing the data of around 73 million insured persons per year. The data starts in 2008, with improvements data availability in 2019. For our analysis we have considered the broader data extent from 2019. Data stems from both primary and secondary care settings, as well as specialist care, as long as reimbursed through public health insurance. |
| Cancer registry – Epidemiological | Zentrum für Krebsregisterdaten (Centre for Cancer Registry Data, ZfKD) | The ZfKD bundles the data from all Germany’s state cancer registries. Originally only collecting epidemiological data, the centralized register now also contains clinical data from 2020 onwards. As cancer registration is mandatory in Germany, all diagnosed cancer cases are contained in the register, with the epidemiological information being available from all states since 2007. |
| Cancer registry - Clinical | Klinisches Krebsregister Niedersachsen (Clinical Cancer Registry Lower Saxony, KKN) | The KKN contains information, including clinical data, on all cancer diagnoses in the German state of Lower Saxony. As cancer registration is mandatory in Germany, all diagnosed cancer cases diagnosed in Lower Saxony are contained in the register, starting from July 2018. |
| ALS registry | ALS-Register Schwaben (ALS registry Swabia) | The ALS registry Swabia collects epidemiological and clinical data on ALS cases diagnosed in the German region of Swabia. For some participants, biological samples are also collected. Start of data collection was October 2010, with additional retrospective inclusion of cases diagnosed from October 2008. |
| EHR data | Beobachtungspraxen-Netzwerk Hannover (BeoNet Hannover) | BeoNet-Hannover collects electronic health records from primary care practices across Germany from 2016 onwards. The data source contains pseudonymised data from than 300.000 patients. |

*Table S1: Data source descriptions*

| **Level** | | **Example** |
| --- | --- | --- |
| *Availability – Eligibility Criteria* | | |
| 1 | Data directly available | Use available information in the data source capturing patients’ medication history to replicate the inclusion/exclusion criterion “no prior medication with metformin” |
| 2 | Can be approximated with available data | Use of available data about age and medical history to replicate the inclusion/exclusion criterion "post-menopausal" |
| 3 | Not feasible to approximate | A patient’s height in claims databases |
| *Importance – Eligibility Criteria* | | |
| 1 | Without impact on research question | Administrative criteria like signed informed-consent forms |
| 2 | Conservative estimation possible | Exclusion of patients with heart failure of New York Heart Association (NYHA) grade IV in effectiveness studies. Exclusion of all heart failure patients as an approximation would lead to better or equal performance of the control arm in relevant outcomes |
| 3 | Crucial for research question | Human epidermal growth factor receptor 2 (HER2) status in a trial of a HER2-targeted therapy |
| *Availability – Primary Endpoint* | | |
| 1 | Exactly available | Overall survival in data sources recording the exact date of death |
| 2 | Conservative estimation possible | Measurement of time to treatment discontinuation in RWD sources which often can only be overestimated by recording patients not refilling their prescriptions |
| 3 | Not available | Any endpoint that cannot validly be evaluated in RWD sources, e.g. overall response rates evaluated at a specific time point or monitored continuously |

*Table S2: Definition of availability and importance levels*
